# The Effect of Dietary Patterns on Clinical Pregnancy and Live Birth Outcomes in Men and Women Receiving Assisted Reproductive Technologies: A Systematic Review and Meta-Analysis

**DOI:** 10.1093/advances/nmac023

**Published:** 2022-03-16

**Authors:** Nicole J Kellow, Jake Le Cerf, Fabrizzio Horta, Aimee L Dordevic, Christie J Bennett

**Affiliations:** Monash University Department of Nutrition, Dietetics, and Food, Melbourne, Victoria, Australia; Monash University Department of Nutrition, Dietetics, and Food, Melbourne, Victoria, Australia; Monash Health Department of Obstetrics and Gynecology, Melbourne, Victoria, Australia; Monash IVF, Clayton, Melbourne, Australia; Monash University Department of Nutrition, Dietetics, and Food, Melbourne, Victoria, Australia; Monash University Department of Nutrition, Dietetics, and Food, Melbourne, Victoria, Australia

**Keywords:** fertility, pregnancy, nutrition, dietary patterns, assisted reproductive technology

## Abstract

The nutritional status of reproductive-aged couples can have a significant impact on fertility status, but the effect of dietary patterns on pregnancy outcomes in people using assisted reproductive technologies (ARTs) is currently unknown. This review aimed to synthesize the published research investigating the relation between preconception dietary patterns and clinical pregnancy or live birth in men and women of reproductive age undergoing ART. Six electronic databases were systematically searched for original research published between January 1978 and June 2021. Original research reporting on the effect of predefined dietary patterns on either clinical pregnancy and/or live birth rates following in vitro fertilization (IVF) or intracytoplasmic sperm injection (ICSI) in men and women aged 18–49 y was eligible for inclusion. Studies were assessed for risk of bias according to the Cochrane guidelines. Included studies underwent qualitative and quantitative synthesis using random-effects model meta-analyses. Thirteen studies (12 cohort studies, 1 randomized controlled trial) reporting on 3638 participants (93% female) were included in the review. All studies had a moderate–high risk of bias. In individual studies, maternal adherence to 4 dietary patterns [Mediterranean diet (RR: 1.22; 95% CI: 1.05, 1.43), novel profertility diet (OR: 1.43; 95% CI: 1.19, 1.72), Iranian traditional medicine diet (OR: 3.9; 95% CI: 1.2, 12.8), Dutch national dietary recommendations diet (OR: 1.65; 95% CI: 1.08, 2.52)] was associated with increased likelihood of achieving a clinical pregnancy, while 2 dietary patterns [novel profertility diet (OR: 1.53; 95% CI: 1.26, 1.85), Mediterranean diet (RR: 1.25; 95% CI: 1.07, 1.45)] were associated with increased probability of live birth. Meta-analyses showed an association between adherence to the Mediterranean dietary pattern and live birth across 2 studies (OR: 1.98; 95% CI: 1.17, 3.35; *I*^2^ = 29%, *n* = 355), but no association with clinical pregnancy. As the relation between dietary patterns and ART outcomes is currently inconsistent, higher-quality nutrition research is required to further explore this emerging field of interest (PROSPERO registration: CRD42020188194).

## Teaser

**Statement of Significance**: The association between dietary patterns and ART-assisted fertility outcomes is currently inconsistent, but a small number of dietary patterns show promise and warrant further investigation. To improve the quality of future nutrition research exploring fertility outcomes, habitual dietary intake should be regularly assessed using validated FFQs and statistical analyses should include adjustment for age, BMI, total energy intake, and the dose and frequency of nutritional supplement use.

## Introduction

Infertility is defined as the inability to become pregnant after 12 mo of regular, unprotected sexual intercourse (1). The inability to successfully conceive a child or carry a pregnancy to term can have devastating long-term social, psychological, and financial consequences for infertile couples (2). It is estimated that up to 15% of all couples of reproductive age worldwide suffer from infertility (3). Male factor infertility contributes to 20–30% of all infertility cases, while female factor infertility may be the sole factor in up to 30% of cases, and approximately 30% of cases are of unknown etiology or are attributable to a combination of male and female factors (1, 4).

Infertile couples are increasingly utilizing assisted reproductive technology (ART) to improve their chances of conception (5). ART is the collective term that describes the medical and pharmacological methods used to treat infertility and includes in vitro fertilization (IVF) and intracytoplasmic sperm injection (ICSI). As only approximately 30–40% of couples attempting ART will achieve a live birth with each treatment cycle (6), researchers continue to investigate complementary factors that may increase the likelihood of successful outcomes.

Over the last decade, there has been increased scientific interest in the effect of modifiable preconception dietary and lifestyle factors on ART outcomes (7). As dietary intake of specific micronutrients can influence the concentration of vitamins, minerals, and antioxidants in both follicular and seminal fluid, achieving an optimal maternal and paternal nutritional intake prior to and during ART has been hypothesized to potentiate reproductive success (8, 9). Recent research has found some associations between nutritional intake and both positive and negative clinical outcomes of ART. Nutrients associated with improved fertility in women undergoing ART include folic acid (10), vitamin D (in cases of deficiency) (11), and omega-3 PUFAs (12), while nutritional factors such as overweight and obesity (13), and the overconsumption of *trans* fat (14), sugar (15) and environmental toxins such as pesticides and heavy metals (16), are associated with reduced fertility following ART. The impact of alcohol and caffeine intake in both men and women during ART remains contradictory (17). A higher dietary antioxidant intake in males undergoing ART has frequently been associated with improved sperm characteristics (18, 19), but some trials providing antioxidant supplements to men undergoing ART have found no such improvements (20).

Typically, studies that have examined the relation between diet and ART outcomes have focused on single nutrients and supplements rather than whole dietary patterns. Most studies have been conducted in small sample sizes, and many have only measured in vitro intermediate outcomes of fertility status (sperm count, sperm quality, oocyte quality) rather than patient-important primary outcomes such as clinical pregnancy and/or live birth. In addition, many trials involving nutritional supplementation provide a combination of different dietary compounds (antioxidants, vitamins, or other nutrients) simultaneously at a wide range of dosages, making it difficult to compare studies or combine similar studies to achieve greater statistical power (21). The intake of individual nutrients is likely to have limited relevance to the study of ART outcomes because people consume whole foods and diets that contain a highly complex combination of nutrients and anti-nutrients, which interact to either enhance or impair the absorption of numerous dietary components. Dietary patterns are now the focus of increasing research efforts, and it is important that the diet as a whole is considered when examining its impact on health outcomes.

This review aims to synthesize the body of published research investigating the relation between preconception dietary patterns and clinical pregnancy and/or live birth outcomes in men and women of reproductive age undergoing ART, to determine whether an optimal dietary pattern for ART can be identified. In addition, strengths and limitations of the current evidence will be identified and recommendations will be made for improving the quality of nutritional research in this emerging field.

## Methods

### Eligibility criteria, information sources, search strategy

This review was conducted according to the Preferred Reporting Items for Systematic Reviews and Meta-Analyses guidelines (PRISMA) (22) and was registered with the Prospective Register of Systematic Reviews (PROSPERO; registration number CRD42020188194) on 5 July 2020.

This review sought articles from the following research databases: Ovid Medline, Scopus, CINAHL Plus, Embase, Web of Science, and the Cochrane Database of Systematic Reviews. The databases were searched for literature published between 1 January 1978 to 1 June 2021. January 1978 was selected as the start date for the literature search as this was the year of the first live birth resulting from IVF (23).

Eligible study designs included observational, cross-sectional, cohort, clinical trial, and randomized clinical trials. Eligible participants included both males and females, aged 18–49 y, who were undergoing IVF or ICSI. Studies were required to assess dietary patterns via prospective collection of dietary intake data using FFQs or via retrospective dietary recall methods and stratification into specific dietary patterns. Primary outcomes considered important for the purposes of this review were rates of clinical or biochemical pregnancy and/or live birth following ART.

The search terms used were as follows: (“reproductive techniques” OR “reproductive techniques, assisted” OR “assisted reproducti*” OR “in vitro fertili*” OR “invitro fertili*” OR “IVF” OR “test tube babies” OR “test tube baby” OR “intracytoplasmic sperm injection*” OR “ICSI”) AND (“diet” OR “feeding behaviour” OR “diet, food, and nutrition,” “nutrition* adj3 (therap* or supplement* or intervention* or plan* or prescri* or educat* or program or advice or support or replacement or substitut* or pattern* or intake or habit)”) OR (“diet* adj3 (therap* or supplement* or intervention* or plan* or prescri* or educat* or program or advice or support or replacement or substitut* or pattern* or intake or habit)”) OR (“food* or vitamin* or mineral* or diet* or drink or beverage”) AND (“pregnancy” OR “pregnan*” OR “live birth*” OR “still birth or stillbirth or still born or stillborn or miscarr*” OR “abortion”). The search was then limited to studies in human participants, and those published in English. An example of the search strategy used is included in **Supplemental Table 1**.

Studies were excluded if their primary analysis concerned consumption of individual foods, food groups, vitamins, or minerals, as opposed to dietary patterns. Studies that examined proxy estimates of fertility status such as sperm or oocyte morphology, quality, or quantity were not included because these outcomes often correlate poorly with pregnancy and live birth rates. Ecological and in vitro study designs were not included. Studies that specifically recruited women with conditions associated with subfertility such as polycystic ovarian syndrome or endometriosis were excluded. However, studies involving large numbers of reproductive-aged women (which may have included some women with these conditions) were included in this review. The reference lists of included studies were searched manually to supplement the electronic search strategy.

### Study selection

All references resulting from the search process were collated and uploaded to Endnote (version 9.2; Clarivate Analytics). Following removal of duplicate manuscripts, all remaining studies were imported into the Covidence screening software (Covidence Systematic Review Software; Veritas Health Innovation). All articles underwent title and abstract screening, with included articles then progressing to full-text screening. Both the title and abstract and the full-text screening were independently completed by 2 authors (NJK, JLC). Conflicts that arose in each screening phase were resolved by discussion between these authors until consensus was achieved.

### Data extraction

Data extraction was undertaken following full-text screening. Data collected included first author and year of publication, country of origin, study design, number of participants, participant characteristics, intervention or dietary patterns assessed, method of dietary assessment, outcome(s) measured, associations between dietary pattern, and fertility and adjustments made for covariates. One author conducted the data extraction (NJK), which was then verified by a second author (JLC).

### Risk-of-bias assessment

Risk-of-bias assessment was undertaken independently by 2 authors (NJK, JLC). Cohort studies were assessed using the Risk Of Bias In Non-randomized Studies–of Interventions (ROBINS-I) tool (24). This tool identifies potential biases within studies based on a set of 7 domains, including confounding, selection of participants, classification of interventions, deviation from intended interventions, missing outcome data, outcome measurement bias, and selection of reported result. The risk of bias for each study was classified as either “no information,” “low risk,” “moderate risk,” “serious risk,” or “critical risk.” The randomized controlled trial (RCT) was assessed for risk of bias using the Cochrane Risk-of-Bias 2.0 tool for randomized trials (RoB 2.0) (25). This tool evaluates potential biases within studies based on a set of 5 domains, including bias arising from the randomization process, deviations from intended interventions, incomplete outcome data, outcome measurement bias and selective reporting of results. Risk of bias for the RCT was designated as either “low risk,” “some concerns,” or “high risk.” Inconsistencies between the reviewer's risk-of-bias assessments at the study level were resolved through discussion until consensus was reached.

### Data synthesis

Where studies assessed clinical pregnancy and live birth outcomes with consumption of similar dietary patterns, that dietary pattern was subjected to random-effects model meta-analyses using Review Manager (RevMan, version 5.1; The Nordic Cochrane Centre, The Cochrane Collaboration, 2014, Copenhagen, Denmark). Due to limited concordance between the various scoring systems used to assess adherence to the Mediterranean diet (26), only studies using the same adherence assessment tool were pooled for analysis. ORs were determined for each outcome with 95% CIs, either where the highest category of adherence was compared with the lowest category of adherence (reference group) or where the highest category of adherence was determined to be the reference group. Data were collected from the model that adjusted for the highest number of potential confounders. Results were combined for each fertility outcome and data were tested for interstudy heterogeneity using the Cochrane Q statistic and quantified by the *I*^2^ statistic with statistical significance defined as *P* < 0.05.

## Results

### Study selection

**FIGURE 1:**
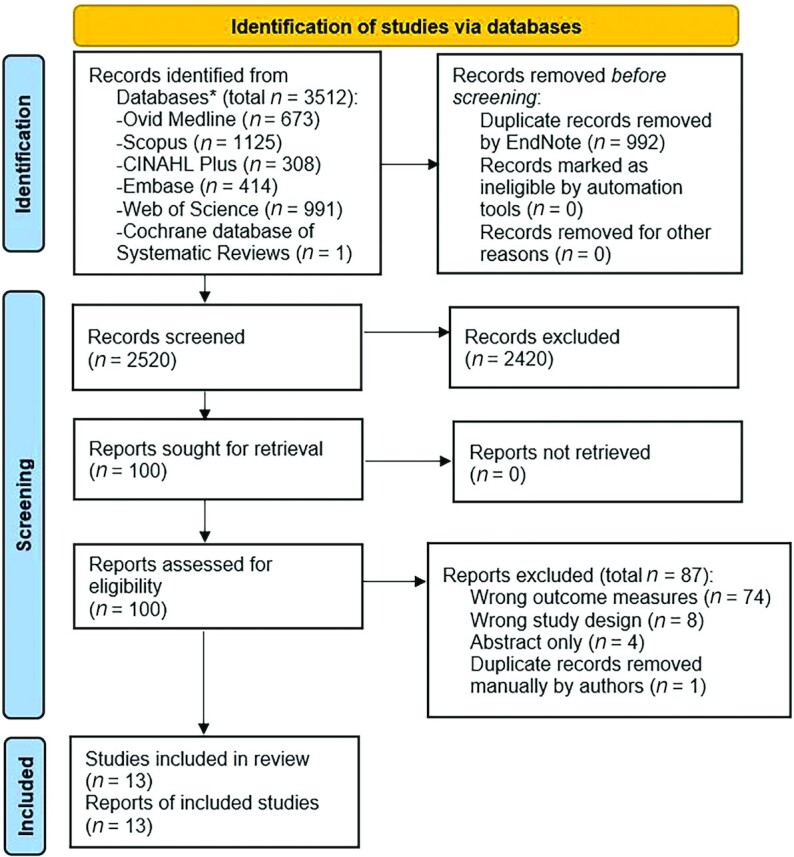
PRISMA flowchart detailing progression of studies through the review process. *Hand-searching of the reference lists of the included studies was undertaken but no additional studies were identified. PRISMA, Preferred Reporting Items for Systematic Review and Meta-Analyses.

Initial database searches yielded a total of 3511 citations. Following the removal of duplicate articles and studies that did not meet the inclusion criteria, 13 studies (27–39) were available for qualitative synthesis, 4 of which were available for quantitative analysis (30, 32, 35, 37). The PRISMA flowchart depicting the study selection process is shown in **Figure 1**.

### Study characteristics

Characteristics of the 13 included studies are detailed in **Table 1**. All studies were published between 2010 and 2020, and collectively contained 3638 participants. Most studies primarily recruited women undergoing IVF or ICSI (*n* = 3388), with the exception of Braga and colleagues (28) who exclusively studied males participating in ICSI (*n* = 250). Although primarily focused on data collection from female study participants, 2 studies also collected dietary information from male partners (38, 39). Twelve of the 13 studies used a prospective cohort study design, while Alibeigi and colleagues (27) conducted a randomized controlled clinical trial (prospectively registered in the Iranian Center for Clinical Trials, code no. IRCT2017013032245N2). Three of the included studies were conducted in Iran (27, 31, 33), with 2 studies each conducted in Brazil (28, 29), The Netherlands (38, 39), and Italy (34, 35), whereas single studies were completed in the United States (30), Greece (32), Japan (36), and China (37).

**TABLE 1:** Characteristics of included studies investigating the relation between preconception dietary patterns and clinical pregnancy and/or live birth outcomes in men and women of reproductive age undergoing ART^1^

First author (year of publication) (ref)	Country of origin, study design	Number of participants, participant characteristics	Length of study, duration of follow-up	Intervention or dietary pattern(s)	Method of dietary assessment	Rate of follow-up, %	Outcome(s) measured	Association between dietary pattern and fertility	Adjustment for covariates and confounding factors
Alibeigi (2020) (27)	Iran, randomized clinical trial	86 women undergoing IVF randomly assigned to either control group (*n* = 45) or intervention group (*n* = 41), age range: 20–40 y; mean age ± SD: 30.1 ± 4.5 y; mean BMI ± SD: 25.2 ± 4.4 kg/m²	Minimum 3-mo intervention period; regular follow-up of participants (once/fortnight)	Control group: received “modern dietary recommendations” (details not provided). Intervention group: received advice to follow “Iranian traditional medicine diet and lifestyle.” Recommended foods included lamb, chicken, quail, shrimp, fish (limited amount), rice, chickpeas, beans, barley, wheat, bread, milk, honey, eggs, olive oil, animal butter, pomegranate sauce, cinnamon, saffron, cloves, and cooked vegetables (onions, garlic, apples, carrots, acanthus, pumpkin, okra). Foods/activities not allowed included overeating, eating several foods in a meal (no food mixing allowed), drinking liquids with or immediately after food, fast food, canned food, foods with preservatives, frozen foods, beef, camel meat, rhubarb, celery, radish, turnip, cabbage, eggplant, tuna, mayonnaise, tomato paste, pasta, lentils, mushrooms, processed meats, iced water, or sour foods/drinks. Intervention group also joined a participant discussion group	Completion of FFQ at baseline (type not specified) and multiple 24-h dietary recalls during the study to assess adherence (methodology not specified)	100%	Chemical/clinical pregnancy (defined as an elevated serum β-hCG level of >25 mIU/mL)	Sixty percent of participants (*n* = 12/20) in the intervention group achieved a chemical/clinical pregnancy compared with 15% of participants (*n* = 5/35) in the control group; OR: 3.9; 95% CI: 1.2, 12.8; *P* = 0.022. Maternal age had a significant effect on IVF outcome, with pregnancy occurring more frequently in women of a younger age. The mean age ± SD of participants with successful and unsuccessful IVF outcomes was 27.94 ± 3.65 y and 31.57 ± 4.24 y, respectively (*P* = 0.002)	Adjusted for level of education and number of previous IVF attempts. No significant difference in dietary macro- or micronutrient intakes between the 2 groups
Braga (2015) (29)	Brazil, prospective cohort	269 women undergoing ICSI (baseline characteristics not reported)	January 2012 to July 2013 (17 mo)	Frequency of consumption from the following food categories was assessed: cereals, vegetables, legumes, fruits, red meat and pork, chicken, fish, dairy products, chocolate, soft drinks, caffeine-containing soft drinks, alcoholic drinks, dietary sweetener, and coffee	Baseline completion of a validated FFQ (modified by the researchers)	Not stated	Clinical pregnancy (defined as the presence of fetal heart activity by ultrasound at 6 to 7 wk of gestation after embryo transfer)	The consumption of red meat (OR: 0.68; 95% CI: 0.48, 0.89; *P* = 0.042), increased BMI (OR: 0.43; 95% CI: 0.25, 0.93; *P* = 0.046), and being on a weight-loss diet (OR: 0.79; 95% CI: 0.56, 0.97; *P* = 0.016) were negatively associated with the likelihood of clinical pregnancy	Adjusted for maternal age, number of retrieved oocytes, and fertilization rate
Braga (2012) (28)	Brazil, prospective cohort	250 men undergoing ICSI; mean age ± SD: 38.4 ± 9.3 y; mean BMI ± SD: 26.9 ± 4.4 kg/m²; mean age ± SD of female partner: 32.3 ± 4.4 y	Not stated	Frequency of consumption from the following food categories was assessed: cereals, vegetables, legumes, fruits, red and pork meat, chicken, fish, dairy products, sweet foods, alcoholic drinks, caffeine-containing soft drinks, and coffee	Baseline completion of validated FFQ (modified by the researchers)	Not stated	Clinical pregnancy (defined as the presence of fetal heart activity by ultrasound at 6–7 wk of gestation), miscarriage rate (spontaneous pregnancy loss before 24 wk gestation)	Clinical pregnancy negatively influenced by meat consumption (OR: 0.06; 95% CI: 0.06, 0.7; *P* = 0.042), being on a weight-loss diet (OR: 0.21; 95% CI: 0.01, 1.19; *P* = 0.011), and female partner's BMI (OR: 0.43; 95% CI: 0.25, 1.13; *P* = 0.027). Although the *P* values for being on a weight-loss diet and female partner's BMI are significant, the 95% CIs are not. Rate of miscarriage was not influenced by any food consumption or social habit	Adjusted for maternal and paternal age, number of retrieved oocytes, number of transferred embryos, endometrium thickness, FSH dose, maternal smoking, and female BMI
Gaskins (2019) (30)	USA, prospective Cohort	357 women undergoing IVF/ICSI with mean age ± SD: 35.3 ± 4.0 y and mean BMI ± SD: 24.1 ± 4.3 kg/m^2^	Assessed data from 2007 to 2017	Participant diets were scored for adherence to 4 different dietary patterns: the “Mediterranean Diet Score” (MedDietScore) (range: 0–55) based on dietary intake of 11 items: vegetables, potatoes, legumes, fruit, whole grains, high-fat dairy, red meat, fish, poultry, olive oil, and alcohol. Scoring was reversed for consumption of red meats, poultry, full fat dairy, and excessive alcohol. The “alternate Healthy Eating Index 2010” (range: 0–110) was scored higher on consumption of vegetables (excluding potatoes), fruit, whole grains, nuts and legumes, long-chain omega-3 fats, polyunsaturated fat, and alcohol, while scoring was reversed for higher intake of sugar-sweetened beverages and fruit juice, red and processed	Baseline completion of validated FFQ and calculation of adherence to each dietary pattern. Nutritional supplement type, dose, and frequency of use were also incorporated into calculation of nutrient intake for each participant	Women were followed up for 1 (55%), 2 (26%), 3 (13%), or 4–6 cycles (5%)	Clinical pregnancy (defined as the presence of an intrauterine pregnancy confirmed by ultrasound at 6 wk gestation); live birth (defined as the birth of a neonate on or after 24 wk of gestation)	No significant results found for adherence to the alternate Healthy Eating Index 2010 or the Fertility Diet. Women in the second, third, and fourth quartiles of Mediterranean diet adherence had significantly higher probability of live birth (adjusted proportion: 0.44; 95% CI: 0.39, 0.49; *P* < 0.05) compared with women in the first quartile (adjusted proportion: 0.31; 95% CI: 0.25, 0.39) but there was no additional benefit of adherence to the Mediterranean diet above the second quartile. Women with the highest adherence to the novel	Adjusted for age, energy intake, BMI, smoking status, and moderate to vigorous exercise quantity
				meat, *trans* fat, and sodium. The “Fertility Diet” (range: 8–40) assigned points for the ratio of MUFAs to *trans* fat, percentage of energy from vegetable protein, high-fat dairy, iron, and multivitamins. For percentage of energy from animal protein, glycemic load, and low-fat dairy, the point assignment was reversed. The novel “Profertility Diet” score (range: 9–36) developed by the authors included a higher intake of supplemental folic acid, vitamin B-12, vitamin D, low-pesticide fruits and vegetables, whole grains, seafood, dairy, and soy foods, while scoring was reversed for intake of high pesticide fruits and vegetables				profertility diet (score: 26–32; *n* = 86) demonstrated significantly higher clinical pregnancy and live birth rates. Adjusted proportion for clinical pregnancy was 0.61 (95% CI: 0.52, 0.69; *P* < 0.05), while adjusted proportion for live birth was 0.56; (95% CI: 0.47, 0.64; *P* < 0.05). Across all quartiles, *P*-trend < 0.001 for both clinical pregnancy and live birth rates	
Jahangirifar (2019) (31)	Iran, prospective cohort	140 women undergoing IVF/ICSI; mean age ± SD: 32.4 ± 5.2 y; mean BMI ± SD: 28.1 ± 4.9 kg/m^2^	Not stated	Authors used FFQ and factor analysis to categorize participant diets into either “Healthy dietary pattern,” “Western dietary pattern,” or “Unhealthy dietary pattern.” The “Healthy dietary pattern” included high consumption of fruits, nuts, vegetables, red and white meat, dairy, green olives, cream, and legumes. The “Western dietary pattern” included high consumption of sweet drinks, sweets, caffeinated drinks, potatoes, fast foods, refined grains, liquid oils and salt. The “Unhealthy dietary pattern” included high consumption of mayonnaise, butter, egg, junk foods, and solid oils and low consumption of whole grains	Baseline completion of 168-item validated FFQ	65%	Clinical pregnancy (defined as the presence of 1 or more gestational sacs during transvaginal scan 3 wk after embryo transfer)	Clinical pregnancy only significantly different across tertiles for consumption of the “Unhealthy dietary pattern.” Tertile 1 (lowest intake) was set as the reference tertile. “Unhealthy dietary pattern”: tertile 1 [*n* = 46; OR = 1.00 (reference)] vs. tertile 2 (*n* = 47; adjusted OR: 0.09; 95% CI: 0.01, 0.6;*P*-trend = 0.022). Tertile 3 not significant	Adjusted for age, marriage age, BMI, waist circumference, physical activity, total energy intake, supplement consumption (yes/no), duration of metformin use
Karayiannis (2018) (32)	Greece, prospective cohort	244 women undertaking their first IVF/ICSI treatment (age range: 22–41 y, all with BMI < 30 kg/m^2^)	3 y, 2013 to 2016	Validated Mediterranean Diet Score (MedDietScore) calculated for each participant and their male partner, MedDietScore range = 0–55, where higher values indicate greater adherence to the Mediterranean diet	Baseline completion of a semi-quantitative 76-item FFQ validated for the Greek population	Not stated	Clinical pregnancy (defined as the presence of an intrauterine pregnancy confirmed by ultrasound—presence of at least 1 gestational sac and cardiac activity at 6 wk estimated gestational age); live birth (birth of a neonate on or after 24 wk of gestation); adverse outcomes (e.g., miscarriage) not assessed	Compared with women in the highest tertile of the MedDietScore (MedDietScore ≥36, *n* = 86), women in the lowest tertile (MedDietScore ≤30, *n* = 79) had significantly lower rates of clinical pregnancy (29.1 vs. 50.0%, *P* = 0.01) and live birth (26.6 vs. 48.8%, *P* = 0.01). Adjusted RR for clinical pregnancy comparing women in the lowest with women in the highest tertile of the MedDietScore was 0.35 (95% CI: 0.16, 0.78; *P*-trend = 0.01), and RR for live birth was 0.32 (95% CI: 0.14, 0.71; *P*-trend = 0.01). Significant association between MedDietScore and clinical pregnancy/live birth only observed in women aged <35 y	Adjusted for maternal age, ovarian stimulation protocol, BMI, physical activity, State and Trait Anxiety, infertility diagnosis, total energy intake, and dietary supplements used (frequency and type of supplement)
Kazemi (2014) (33)	Iran, prospective cohort	240 women undergoing IVF; age and BMI not reported	July 2010 to April 2011	Quantification of energy intake and total dietary fat and its components. Components of total fat considered were SFAs, MUFAs, and PUFAs. Individual foods assessed as major fat sources were oil, meat (red meat, fish, chicken), sausage, and turkey ham (as a subgroup of meat) and dairy foods	Baseline completion of 168 item validated FFQ	98.30%	Clinical pregnancy: after a positive biochemical pregnancy test, an ultrasound scan was performed 4 wk later to determine pregnancy	No significant difference found in clinical pregnancy rate between groups with ≤35% energy intake as fat versus >35% energy intake as fat; 30.8% of participants achieved a clinical pregnancy with ≤35% energy intake as fat (*n* = 182) and 34.5% of participants achieved a clinical pregnancy with >35% energy intake as fat (*n* = 54, *P* > 0.05)	Adjusted for age, physical activity, BMI, and etiology of infertility
Noli (2020) (34)	Italy, prospective cohort	494 women undergoing IVF (mean age ± SD: 36.6 ± 6.0 y; mean BMI ± SD: 22.4 ± 4.0 kg/m^2^)	September 2014 to December 2016	Calculation of GI and GL of participants' diets. For each food containing carbohydrates, the GI was expressed as the percentage of the postprandial glucose response considering white bread as standard food and referring to the published international GI tables. The average daily GI of every subject was calculated by summing the products of the GI of 1 serving of each food multiplied by the average number of servings of that food consumed by the person per week, divided by the weekly available carbohydrates. The daily average GL was computed by summing the products of the GI of 1 serving of each food multiplied by the average number of servings of that food consumed by the subject per week	Baseline completion of 78-item validated FFQ, which assessed the average weekly consumption of food items	95.00%	Clinical pregnancy: defined as the presence of at least 1 intrauterine gestational sac; live birth: defined as the birth of a viable newborn on or after 24 wk of gestation	No significant difference found in clinical pregnancy rate between highest GI quartile (GI >80.1) and lowest GI quartile (GI <74.2): adjusted RR: 1.04; 95% CI: 0.92, 1.17; *P*-trend = 0.89. No significant difference found in live birth rate between highest GL quartile and lowest GL quartile: adjusted RR: 1.02; 95% CI: 0.91, 1.15; *P*-trend = 0.97	Adjusted for age, college degree, BMI, leisure physical activity, and number of previous ART cycles
Ricci (2019) (35)	Italy, prospective cohort	474 women undergoing IVF (mean age ± SD: 36.6 ± 3.6 y, mean BMI ± SD: 22.3 ± 4.0 kg/m^2^)	September 2014 to December 2016	Mediterranean Diet Score (MDS) (range = 0–9) calculated for each participant based on their dietary intake of 9 dietary components: fruit, vegetables, cereals (including bread and potatoes), legumes, fish, MUFA:SFA ratio, dairy products, meat (including meat products), and alcoholic beverages	Baseline completion of 78-item validated FFQ, followed by calculation of Mediterranean Diet Score (MDS)—validated but modified to include potatoes	94.60%	Clinical pregnancy: defined as the presence of at least 1 intrauterine gestational sac; live birth: criteria not described. Tertile 1 was the reference tertile, being 0–3 adherence score. Tertile 2: 4–5 adherence score. Tertile 3: 6–9 adherence score. Stratified into <35 y old and ≥35 y old, as well no as stratification – overall sample result	Reported exclusively on data obtained from the female partner; no significant changes observed in clinical pregnancy rate or live birth rate in any stratification (all *P* > 0.05). Adjusted RR of tertile 1 was 1.00 (reference tertile, Mediterranean Diet Score: 0–3; *n* = 132). Tertile 3 (Mediterranean Diet Score: 6–9, *n* = 142) clinical pregnancy adjusted RR: 0.98; 95% CI: 0.87, 1.09; *P*-trend = 0.68. Live birth rate adjusted RR: 0.99; 95% CI: 0.89, 1.11; *P*-trend = 0.87	Adjusted for maternal age, leisure physical activity, BMI, smoking, daily energy intake, and number of previous IVF cycles
Sugawa (2018) (36)	Japan, prospective cohort	140 women undergoing IVF; mean age ± SD: 37 ± 4.2 y; mean BMI ± SD: 21.1 ± 2.9 kg/m^2^	November 2016 to December 2016	Factor analysis was used to identify and score participant adherence to 3 dietary patterns: the “Healthy/vegetables and seafood” dietary pattern, the “Western” dietary pattern, or the “Rice and miso soup” dietary pattern. The “Healthy/vegetables and seafood” dietary pattern had a high factor loading for vegetables, fish, seafood, soy products, and chicken. The “Western” dietary pattern had a high-positive factor loading for red meat, chicken, and oils and a high negative loading for seafood. The “Rice and miso soup” dietary pattern had a high-positive factor loading for rice and miso soup and a negative loading for confectionery. Overall, the 3 dietary patterns accounted for 26.6% of the variance in food intake	Baseline completion of 58-item FFQ validated for the Japanese population	Not stated	Clinical pregnancy: defined by detection of an intrauterine gestational sac by ultrasound scan ∼21 d after egg retrieval	No significant differences in clinical pregnancy across quartiles for any dietary pattern (*P*-trend > 0.05), where Q1 = reference quartile, Q4 = highest adherence to dietary pattern	Adjusted for age, BMI, energy intake, parity, educational level, smoking, alcohol consumption, and folate supplement use (yes/no)
Sun (2019) (37)	China, prospective cohort	590 women undergoing IVF; mean age ± SD: 31.78 ± 3.72 y; mean BMI ± SD: 21.09 ± 2.79 kg/m² (low adherence group) and mean BMI ± SD: 21.15 ± 2.71 kg/m^2^ (high adherence group)	September 2016 to December 2017	Mediterranean Diet Score (MDS) (range = 0–9) calculated for each participant based on their dietary intake of 9 dietary components: fruit, vegetables, bread/cereals, legumes, fish, MUFA:SFA ratio, dairy products, meat (including meat products), and alcoholic beverages. One point usually allocated for moderate alcohol consumption, but authors removed this component, resulting in an adherence score range between 0 and 8	Baseline completion of 69-item nonvalidated FFQ, followed by calculation of Mediterranean Diet Score (MDS) (with removal of the alcohol consumption component)	Not stated	Clinical pregnancy: no definition provided	Higher adherence (*n* = 228, MedDietScore 3–6); lower adherence (*n* = 362, MedDietScore 0–2). Clinical pregnancy rates: higher adherence, 42.62%; lower adherence, 50.94%; *P* = 0.300. No significant differences found	Adjusted for age, duration of infertility, and BMI
Twigt (2012) (38)	The Netherlands, prospective cohort	193 women after IVF/ICSI, *n* = 51 with ongoing pregnancy, *n* = 142 without ongoing pregnancy; median age (IQR) of women with ongoing pregnancy: 32.5 y (30.2, 35.3); median (IQR) age of women without ongoing pregnancy: 33.6 y (29.7, 38.7), *P* = 0.06	October 2007 to October 2010	Adherence to 6 food/dietary categories based on Netherlands Nutritional Centre guidelines, used by authors to calculate PDR score, where increasing PDR score indicated increased dietary quality and adherence to Dutch National Dietary Guidelines. Participants responded to 6 nutritional questions (yes/no): ≥4 servings of whole-grain bread or cereal per day, the use of monounsaturated or polyunsaturated oils, ≥200 g vegetables daily, ≥ 2 pieces of fruit daily, ≥3 servings of meat or meat replacers per week, ≥1 serving of fish per week	Baseline completion of 6 nutritional questions to enable calculation of PDR score. Questions not validated but based on Dutch government dietary guidelines	46.20%	Ongoing pregnancy: defined as a pregnancy with positive fetal heart action at ∼10 wk after embryo transfer confirmed by ultrasound	A 1-point increase in maternal PDR score was associated with a 65% increased odds of ongoing pregnancy (OR: 1.65; 95% CI: 1.08, 2.52; *P* = 0.02). Paternal PDR score not related to ongoing pregnancy (OR: 0.95; 95% CI: 0.48, 1.86; *P* = 0.88). Significant difference found between ongoing pregnancy and no-pregnancy groups on comparison of male partners' whole-wheat intake (male whole-wheat intake was higher in the no-pregnancy group, *P* = 0.03), but no other significant results were found related to individual PDR contributors (all *P* > 0.05)	Adjusted for age, maternal smoking, PDR score of the male partner, BMI of the couple, and treatment indication
Vujkovic (2010) (39)	The Netherlands, prospective cohort	161 women undergoing IVF/ICSI; median age: ∼35 y and median BMI ∼23 kg/m^2^	September 2004 to January 2007	Diets of participants (and their male partners) were classified as belonging to 1 of 2 dietary patterns: “Health conscious-low processed” dietary pattern was characterized by high intakes of fruits, vegetables, whole grains, fish, and legumes, but low intakes of mayonnaise, snacks, and meat products. The “Mediterranean” dietary pattern was characterized by high intakes of vegetable oil, fish, legumes, and vegetables but low intakes of snacks	Baseline completion of 195-item FFQ with principal component analysis used to generate the 2 dietary patterns. FFQ validated for intakes of energy, B-vitamins, and fatty acids	Not stated	Biochemical pregnancy: urine test 15 d after oocyte retrieval	No significant difference in biochemical pregnancy found across tertiles of adherence to either dietary pattern in women: Health conscious-low processed *P*-trend = 0.66 across tertiles, Mediterranean *P*-trend = 0.75 across tertiles. High adherence of the couple to the Mediterranean diet (average adherence score of both partners) increased the probability of pregnancy (OR: 1.4; 95% CI: 1.0, 1.9), although not statistically significant. Dietary data for males not provided in manuscript	Adjusted for age, BMI, smoking, alcohol use, vitamin use (yes/no), treatment type, and stimulation scheme

1 ART, assisted reproductive technology; FSH, follicle-stimulating hormone; GI, glycemic index; GL, glycemic load; ICSI, intracytoplasmic sperm injection; IQR, interquartile range; IVF, in vitro fertilization; PDR, preconception dietary risk; β-hCG: β-human chorionic gonadotrophin.

Primary outcome measures for the purposes of this review were clinical pregnancy and live birth rates. Most studies determined clinical pregnancy by using either blood or urine testing followed by ultrasound detection of an intrauterine gestational sac 3–6 wk post–embryo transfer or detection of fetal heart activity at 6–7 wk estimated gestational age. Twigt and colleagues (38) assessed “ongoing pregnancy,” which they defined as fetal heart activity detected by ultrasound 10 wk following oocyte retrieval. Live birth was defined as the birth of a viable newborn on or after 24 wk of gestation.

### Participant characteristics

Due to the recognized adverse effects of increasing maternal age (40) and BMI (41) on reproductive outcomes, 4 studies included participants within a prespecified age range. The trial by Alibeigi and colleagues (27) recruited women aged 20–40 y, while Gaskins and colleagues (30) included women aged between 18 and 46 y in their analysis. Studies by Karayiannis et al. (32) and Sun et al. (37) both restricted participant age to less than 41 y, and both excluded women with a BMI (in kg/m^2^) ≥30. During statistical analyses, most authors adjusted for the effect of maternal age and BMI on fertility outcomes, with the exception of Alibeigi et al. (27) (where adjustment was not required due to the RCT study design) and Braga et al. (29) (who did not adjust for maternal BMI).

Three studies restricted study inclusion to women with primary infertility only (31–33), and 3 excluded participants receiving donor oocytes (32, 33, 39). Alibeigi et al. (27), Jahangirifar et al. (31), and Kazemi et al. (33) excluded participants with male factor infertility. Three studies excluded women with hydrosalpinx (27, 36, 39), and 3 excluded women with endometriosis (32, 36, 39). In contrast, Alibeigi et al. (27) included women with diagnosed endometriosis. Karayiannis and colleagues (30) excluded participants with any previous IVF attempt. Four studies excluded participants who had made changes to their dietary pattern within the previous 3–12 mo (31–33, 37). Alibeigi et al. (27) and Jahangirifar et al. (31) excluded alcohol consumers and cigarette smokers from study participation.

### Dietary patterns and dietary assessment methods

Sixteen distinct dietary patterns were identified and investigated for their association with clinical pregnancy and/or live birth outcomes during ART. A summary of the characteristics of each dietary pattern is included in Table 1. Five studies explored the association between adherence to the Mediterranean diet and clinically relevant reproductive outcomes (30, 32, 35, 37, 39), whereas 2 studies investigated adherence to a Western dietary pattern (31, 36). Four studies defined novel dietary patterns that had not previously been studied for their influence on fertility: the Iranian traditional medicine diet (27), the novel profertility diet (30), a vegetable and seafood diet (36), and a diet based on Dutch national dietary recommendations (38).

Twelve of the 13 included studies used an FFQ to quantify dietary intake of participants at study baseline. Rather than administering an FFQ, Twigt and colleagues (38) used a 6-item questionnaire with yes or no answers, which was not validated but based on adherence to the Dutch national dietary recommendations. Most studies attempted to utilize validated FFQs, although some authors made modifications to the questionnaires, which may have adversely affected their validity (28, 29). Alibeigi and colleagues (27) did not describe the FFQ used in their trial, or whether it had been previously validated. Sun et al. (37) created a novel FFQ with assistance from their university hospital nutrition department, which was not validated. All 12 of the cohort studies administered the FFQ at baseline only, so individuals making significant dietary changes during the study were not detected. During their RCT, Alibeigi et al. (27) conducted regular 24-h dietary recalls to assess participant compliance, but the methodology used to conduct these recalls was not described. During statistical analyses, 5 studies adjusted for participants’ total energy intake (30–32, 35, 36), and only 4 studies adjusted for the use of nutritional supplements (31, 32, 36, 39). The most comprehensive consideration of nutritional supplement use was undertaken by Gaskins et al. (30), who collected information regarding supplement type, dose, and frequency of use at baseline and incorporated this into the calculation of each participant's nutrient intake.

### Risk of bias of included studies

**FIGURE 2:**
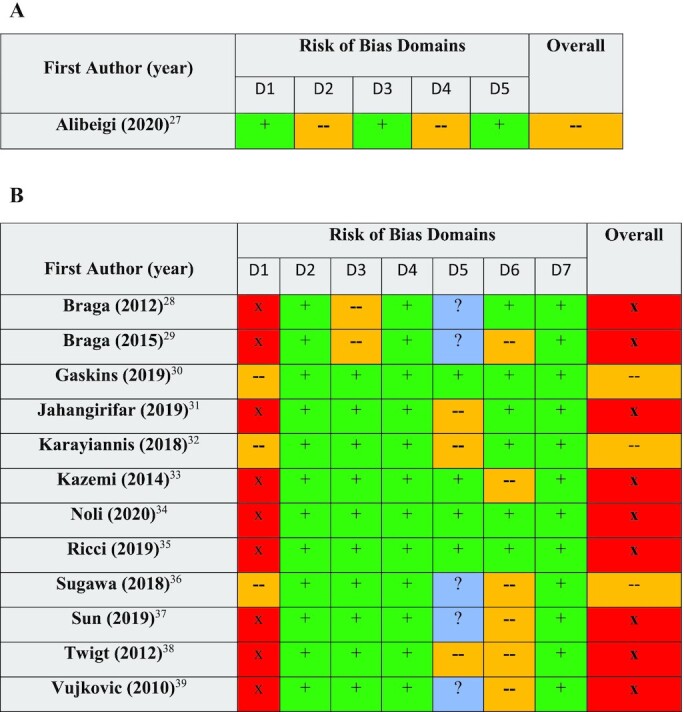
Risk-of-bias assessment of included RCT (A) and non-RCT (B) articles using the ROB2.0 and ROBINS-I tools, respectively. For ROB2.0: D1, Bias arising from the randomization process; D2, Bias due to deviations from intended interventions; D3, Bias due to missing outcome data; D4, Bias in measurement of the outcome; D5, Bias in selection of the reported result. For ROBINS-I: D1, Bias due to confounding; D2, Bias due to selection of participants; D3, Bias in classification of interventions; D4, Bias due to deviations from intended interventions; D6, Bias in measurement of outcomes; D7, Bias in the selection of the reported result. Green “+” = low risk of bias, yellow “—" = moderate risk of bias, red “x” = high risk of bias, and blue “?” = no information. RCT, randomized controlled trial; ROB2.0, Cochrane Risk of Bias 2.0; ROBINS-I, Risk Of Bias In Non-randomized Studies–of Interventions.

Of the 13 included studies, 4 received a “moderate” risk-of-bias rating using either the Cochrane RoB 2.0 or ROBINS-I tools, while the remaining 9 received a “high” risk-of-bias rating (**Figure 2**). The main methodological limitations of the studies included failure to adjust for major study confounders, failure to report study retention rates, and failure to disclose whether outcome assessors or statisticians were blinded.

### Synthesis of results

#### Dietary patterns and clinical pregnancy

Sixteen dietary patterns were investigated in 13 studies for their association with clinical pregnancy outcomes. Four individual studies found significant positive relations between dietary patterns and clinical pregnancy (27, 30, 32, 38), while 1 study reported a significant negative association (31) (Table 1). Remarkably, Alibeigi and colleagues (27) reported that clinical trial participants randomly assigned to follow the Iranian traditional medicine diet and lifestyle demonstrated a 20.9% spontaneous pregnancy rate compared with 2.2% in the control group (OR: 11.5; 95% CI: 2.6, 50.9) prior to commencement of IVF. Of the women who then proceeded to IVF treatment, 60% (12/20) of those following the Iranian traditional medicine diet became pregnant compared with 15% (5/35) of the participants following the control diet.

Gaskins et al. (30) found that each SD increase in maternal adherence to the novel profertility diet was associated with a 43% (95% CI: 19%, 72%) increase in the adjusted odds of clinical pregnancy. This was mediated by significant increases in the probability of successful embryo implantation and significant reductions in the rate of clinical pregnancy loss during the study.

For every 1-point increase in the maternal preconception dietary risk (PDR) score (to indicate adherence to the Dutch national dietary recommendations), Twigt and colleagues (38) reported a 65% increase in the probability of ongoing pregnancy after the first IVF/ICSI treatment. The PDR score of the male partner had no effect on pregnancy outcome.

While Karayiannis et al. (32) found a significant association between Mediterranean diet adherence and clinical pregnancy in women aged <35 y (adjusted RR: 1.22; 95% CI: 1.05, 1.43), studies by Gaskins et al. (30), Ricci et al. (35), Sun et al. (37), and Vujkovic et al. (39) found no such relation. Interestingly, while Vujkovic and colleagues (39) reported no association between maternal Mediterranean dietary intake and clinical pregnancy (OR: 1.02; 95% CI: 0.40, 2.61), when the average Mediterranean diet adherence score was calculated for each woman and her male partner, the couple-level score increased the odds of clinical pregnancy (OR: 1.4; 95% CI: 1.0, 1.9), although this was still not statistically significant.

Jahangirifar and colleagues (31) found a negative association between maternal consumption of an “unhealthy dietary pattern” (characterized by high intakes of mayonnaise, butter, egg, junk foods, and solid oils) and adjusted odds of clinical pregnancy during ART (OR: 0.09; 95% CI: 0.01, 0.6), but this result was only statistically significant between the first and second tertiles. Although the characteristics of a weight-reduction diet were not defined, Braga and colleagues found a cross-sectional association between “being on a weight-loss diet” and reduced odds of achieving a clinical pregnancy in both women (OR: 0.79; 95% CI: 0.56, 0.97) (29) and men (OR: 0.21; 95% CI: 0.01, 1.19) (28) undertaking ICSI treatment.

**FIGURE 3:**
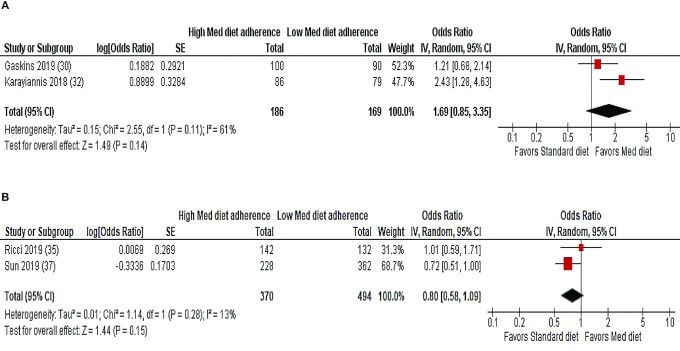
Random-effects meta-analysis of association between maternal adherence to a Mediterranean dietary pattern and clinical/biochemical pregnancy following ART. ORs (95% CI) shown for individual and pooled trials. (A) Studies using the MedDietScore to calculate dietary adherence. (B) Studies using the MDS to calculate dietary adherence. ART, assisted reproductive technology; IV, inverse variance; MDS, Mediterranean Diet Score; Med, Mediterranean.

Meta-analyses of 2 studies using the MedDietScore (30, 32) and 2 studies using the Mediterranean Diet Score (MDS) (35, 37) to explore the association between maternal adherence to a Mediterranean dietary pattern and clinical pregnancy are shown in **Figure 3**. There were no significant associations between pretreatment Mediterranean dietary adherence and the odds of clinical pregnancy for the pooled studies using the MedDietScore (OR: 1.69; 95% CI: 0.85, 3.35; *I*^2^ = 61%; *n* = 355) or the MDS (OR: 0.80; 95% CI: 0.58, 1.09; *I*^2^ = 13%; *n* = 864).

#### Dietary patterns and live birth

The association between adherence to 5 dietary patterns and live birth outcomes was explored in 4 studies (30, 32, 34, 35) (Table 1). Karayiannis and colleagues (32) reported a significant relation between increasing pretreatment adherence to the Mediterranean diet and live birth rates following IVF/ICSI, but only in women aged <35 y. Participants aged <35 y with the highest Mediterranean diet adherence were significantly more likely to achieve a live birth (adjusted RR: 1.25; 95% CI: 1.07, 1.45) than participants aged ≥35 y (adjusted RR: 1.01; 95% CI: 0.93, 1.11). However, Ricci et al. (35) and Gaskins et al. (30) found no consistent relation between Mediterranean diet consumption and live birth.

Preconception adherence to the novel profertility diet was associated with a significant increase in live birth, where each SD (4 points) increase in adherence to the novel profertility diet was associated with a 53% (95% CI: 26%, 85%) higher adjusted odds of live birth (30). Maternal age and BMI had no effect on the outcome. No significant associations were found between adherence to the alternate Healthy Eating Index 2010, the Fertility diet, or a low-glycemic-index (low-GI)/low-glycemic-load (low-GL) diet and live birth (30, 34).

**FIGURE 4:**
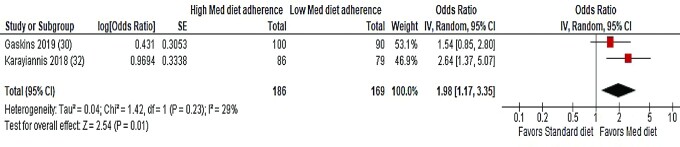
Random-effects meta-analysis of association between maternal adherence to a Mediterranean dietary pattern and live birth following ART. ORs (95% CI) shown for individual and pooled trials. All studies used the MedDietScore to calculate dietary adherence. ART, assisted reproductive technology; IV, inverse variance; Med, Mediterranean.

Two studies (30, 32) using the same Mediterranean diet adherence tool (MedDietScore) were pooled for meta-analysis (**Figure 4**). A significant association was found between adherence to the Mediterranean diet and live birth outcomes (OR: 1.98; 95% CI: 1.17, 3.35; *I*^2^ = 29%; *n* = 355).

## Discussion

This systematic review aimed to synthesize results from studies that assessed the relation between dietary patterns and clinically relevant outcomes of IVF/ICSI in men and women of reproductive age. Sixteen dietary patterns were considered for their association with clinical pregnancy following ART, while 5 dietary patterns were examined for their relation with live birth. Collectively, the associations between dietary pattern adherence and the likelihood of achieving a clinical pregnancy or live birth during ART in the included studies were contradictory. A small number of individual studies demonstrated positive relations between dietary patterns and reproductive outcomes, but the majority found no association. Maternal adherence to a Mediterranean dietary pattern was associated with increased odds of pregnancy and live birth in women aged <35 y in 1 study (32), but 4 other studies investigating the Mediterranean diet found no such associations (30, 35, 37, 39). A meta-analysis of 2 studies (30, 32) using the same Mediterranean diet adherence scoring system found a significant relation between the Mediterranean diet and live birth, but no associations were found for clinical pregnancy. Based on individual study results (27, 30, 32, 38), 4 unique dietary patterns (the novel profertility diet, a diet based on Dutch national dietary recommendations, the Mediterranean diet, and the Iranian traditional medicine diet) show promise as potential strategies to improve ART-assisted outcomes; however, all studies had a moderate–high risk of bias and future observational and interventional research is required to confirm their findings.

While the randomized clinical trial (27) exploring the effect of following the Iranian traditional medicine diet on fertility in women undergoing IVF resulted in significantly higher rates of clinical pregnancy compared with controls, there are some limitations of the study to consider. The sample size was small (*n* = 86), researchers were not blinded to the treatment allocation, and the tools and methods used to assess dietary intakes were not adequately described. Members of the intervention group were also able to attend a virtual discussion/support group (which was not offered to control participants). Because there were no significant differences in macro- and micronutrient intakes between intervention and control groups, the substantial nondietary lifestyle recommendations associated with the Iranian traditional medicine diet may have positively influenced the outcome. Among other lifestyle guidelines, the intervention group was encouraged to avoid exposure to pollution, exercise regularly, manage stress, obtain sufficient sleep, and avoid overeating. Additionally, the strict nature of the diet may preclude its use outside of Iran. Forbidden foods such as tuna, pasta, lentils, beef, canned fruit, iced water, cucumber, and mushrooms may be difficult for some individuals to avoid.

Common features of the dietary patterns identified, including the novel profertility diet, the Dutch national dietary recommendations diet, and the Mediterranean diet, may help to explain why adherence to these eating patterns could be beneficial for fertility. The focus of these diets is on minimally processed fruits and vegetables, whole grains, legumes, nuts, fish, and monounsaturated or polyunsaturated oils, while intakes of highly processed foods are limited. The diets are high in B-vitamins (including folate), antioxidants, omega-3 PUFAs, and dietary fiber and low in saturated fat, sugar, and sodium. Health benefits associated with the consumption of these diets include improvements in metabolic health, lower levels of inflammation and oxidative stress, healthier body weight, and increased diversity of the gut microbiota. Similar metabolic implications are associated with decreased sedentary activity (42) and increased sleep (43). An increased antioxidant profile, achieved by consuming a minimally processed diet, is more readily able to defend against exposure to excessive reactive oxygen species (ROS), which can exert deleterious effects on contributors to both male and female reproductive health, including decreased sperm quality and DNA integrity (44) and poorer quality oocytes (45). In contrast, Jahangirifar and colleagues (31) found an association between maternal adherence to an “unhealthy dietary pattern” and reduced odds of clinical pregnancy during ART. Benefits or detrimental effects of diet on reproductive health are unlikely to be derived from a single feature of these diets, but from the collective contribution of multiple dietary components and other lifestyle factors.

While high fruit and vegetable intakes were a prominent feature of the dietary patterns associated with increased frequencies of clinical pregnancy and live birth in this review, vitamin and mineral supplementation was also widespread in the included studies. Only the studies by Gaskins et al. (30) and Karyiannis et al. (32) considered the full range and frequency of nutritional supplements taken by study participants in their analyses, with both finding that increased adherence to fertility-promoting dietary patterns was positively associated with increasing nutritional supplement intake. While almost all studies in this review used routine maternal folic acid supplementation, individuals with the highest adherence to the novel profertility diet consumed supplements containing much higher doses of folic acid than are currently recommended for the prevention of neural tube defects (30). Folic acid supplementation of at least 800 μg/d has been associated with a higher probability of live birth in women undergoing ART, likely due to higher fertilization rates and lower cycle failure rates prior to embryo transfer (10).

The consumption of whole grains, dietary fiber, and a low-GL diet has been associated with increased fecundability in women attempting pregnancy (46), as well as increased endometrium thickness, embryo implantation, and live birth in women receiving IVF (47). Gaskins et al. (30) found that carbohydrate intake was significantly increased across quartiles with increasing adherence to their novel profertility diet, which may have indicated an increased dietary fiber intake across quartiles. One study (34) included in this review investigated the association between consumption of a low-GI and low-GL diet and clinical pregnancy and live birth rates during ART but found no significant relations. A limitation of this study was that the authors calculated dietary GI values based on maternal consumption of carbohydrate-containing foods only, which does not consider the effect of other nutrients such as fat on the GI of mixed meals.

Discrepancies in the outcomes of the studies investigating associations between maternal adherence to the Mediterranean diet and clinical pregnancy/live birth could be explained by the range of different dietary adherence scoring tools utilized. Gaskins et al. (30) and Karayiannis et al. (32) used the MedDietScore (48) (score: 0–55), Ricci et al. (35) and Sun et al. (37) used the MDS with modifications (49) (score: 0–9), and Vujkovic and colleagues (39) used an FFQ with principal component analysis to calculate and assign dietary pattern adherence scores. Variations between these scoring systems and differences in their food group classifications make them very difficult to compare. While statistically significant, the agreement between the MDS and the MedDietScore is considered to be only moderate (65%) (50).

Health benefits associated with the consumption of the Mediterranean diet may be less pronounced in populations living in non-Mediterranean countries, due to differences in the nutrient composition and agricultural or processing methods of foods grown in the region combined with variations in genetic or microbial profiles of populations between countries. Furthermore, the beneficial nondietary components of the Mediterranean lifestyle, including social interaction and adequate sleep and physical activity, may not be replicated in non-Mediterranean countries (51).

While the studies identified in this review explore dietary patterns, a theme emerged regarding environmental pollutants. In the novel profertility diet there was a unique focus on reducing pesticide consumption (30). Over 105 pesticides, either previously or currently in use, have been identified as having endocrine-disruptive properties in humans (52). In a US prospective cohort study, male consumption of high-pesticide fruit and vegetables was associated with poorer semen quality, lower sperm count, and lower percentage of morphologically normal sperm compared with men with the lowest intakes (53). Further research to determine the potential impact of high-pesticide fruit and vegetable intake on clinical fertility outcomes is required.

Interestingly, Braga et al. (28, 29) found a negative association between self-reported “adherence to a weight-loss diet” and the likelihood of achieving clinical pregnancy in both male and female participants (although no assessment of the quality of the weight-loss diets was undertaken). Weight loss for those who live in a larger body is considered by many experts to be best practice (54). However, the results of Braga et al. are supported by a recent review that identified that there may be little to no benefit of weight loss before ART (54). Further, this practice may be considered discriminatory as it reduces the accessibility of treatment based on body weight (54).

### Strengths and limitations

While this review synthesized data exploring the association between dietary patterns and ART-assisted pregnancy and live birth from a large number of individuals (*n* = 3638), only 7% of study participants were male, so the findings of this review cannot be applied to men. The quality of the male partners’ diet could potentially contribute to the success of reproductive outcomes during ART, but the lack of available dietary pattern data for males in this review prevents any conclusions being made on this topic. Additionally, most studies reported fertility outcomes for both IVF and ICSI procedures combined, so it was impossible to determine whether particular dietary patterns were preferentially beneficial for IVF or ICSI individually.

### Conclusions and implications

Future research in this field should seek to utilize similar, validated dietary assessment tools to enable comparability between studies. Quantification of dietary intake should be repeated at regular intervals throughout extended cohort studies to capture significant dietary changes. Analyses should ideally include adjustment for maternal age and BMI, total energy intake, and the dose and frequency of nutritional supplement use. Only 1 (28) of the 13 studies included in this review principally explored the association between the male partner's diet and clinical ART outcomes (7% of primary study participants were male). Given that men with the lowest adherence to a healthy dietary pattern have been found to have significantly lower sperm counts (55) and sperm motility (56, 57), more studies are required to investigate the influence of the male's dietary patterns on clinical pregnancy and live birth outcomes. Importantly, male fertility is an equal contributor to the reproductive success of couples; however, the use of ART has become a popular adjunctive treatment for alleviating male factor infertility without resolving the underlying cause of infertility. The scarcity of research addressing the relation between male dietary patterns and infertility needs to be addressed.

This review has identified certain dietary patterns that may be capable of positively influencing clinical outcomes of fertility treatment. Based on the current body of evidence, however, the association between dietary patterns and ART outcomes is inconsistent. High-quality studies are required to further elucidate the association between dietary patterns and clinical ART outcomes.

## Supplementary Material

nmac023_Supplemental_File

## Data Availability

Data used in this review is available from the corresponding author upon reasonable request.

## Notes

NJK is supported by a National Health and Medical Research Council (NHMRC) Early Career Research Fellowship (APP1164771). No other external funding was sought or received for this review.

Author disclosures: The authors report no conflicts of interest.

Supplemental Table 1 is available from the “Supplementary data” link in the online posting of the article and from the same link in the online table of contents at https://academic.oup.com/advances/.

Abbreviations used: ART, assisted reproductive technology; GI, glycemic index; GL, glycemic load; ICSI, intracytoplasmic sperm injection; IVF, in vitro fertilization; MDS, Mediterranean Diet Score; PDR, preconception dietary risk; PRISMA, Preferred Reporting Items for Systematic Review and Meta-Analyses; RCT, randomized controlled trial; RoB 2.0, Cochrane Risk-of-Bias 2.0; ROBINS-I, Risk Of Bias In Non-randomized Studies–of Interventions.
