# Supplementary material for: The Effect of Dietary Patterns on Clinical Pregnancy and Live Birth Outcomes in Men and Women Receiving Assisted Reproductive Technologies: A Systematic Review and Meta-Analysis
Source: Adv Nutr. 2022 Mar 16;13(3):857–74. doi: 10.1093/advances/nmac023 (PMC9156378; doi:10.1093/advances/nmac023)
Supplement: nmac023_Supplemental_File [file nmac023_supplemental_file.docx]

**Supplementary Table 1.** Example of database search strategy used (CINAHL Plus)

| **#** | **Search term** |
| --- | --- |
| 1 | Exp reproductive techniques/ or exp reproductive techniques, assisted/ |
| 2 | assisted reproducti* |
| 3 | in vitro fertili* or invitro fertili* or IVF |
| 4 | test tube babies or test tube baby |
| 5 | intracytoplasmic sperm injection* or ICSI |
| 6 | exp Diet/ |
| 7 | exp Feeding Behavior/ |
| 8 | exp "diet, food, and nutrition"/ |
| 9 | nutrition* adj3 (therap* or supplement* or intervention* or plan* or prescri* or educat* or program or advice or support or replacement or substitut* or pattern* or intake or habit) |
| 10 | diet* adj3 (therap* or supplement* or intervention* or plan* or prescri* or educat* or program or advice or support or replacement or substitut* or pattern* or intake or habit) |
| 11 | food* or vitamin* or mineral* or diet* or drink or beverage |
| 12 | exp Pregnancy/ |
| 13 | pregnan* |
| 14 | live birth* |
| 15 | still birth or stillbirth or still born or stillborn or miscarr* |
| 16 | abortion |
| 17 | exp animals/ not humans |
| 18 | 1 or 2 or 3 or 4 or 5 |
| 19 | 6 or 7 or 8 or 9 or 10 or 11 |
| 20 | 12 or 13 or 14 or 15 or 16 |
| 21 | 18 and 19 and 20 |
| 22 | 21 not 17 |
| 23 | limit 22 to yr = "1978 -Current" |
